# Vascular mapping of the face: B-mode and doppler ultrasonography study

**DOI:** 10.4317/medoral.20754

**Published:** 2016-01-31

**Authors:** Maria-José Tucunduva, Raul Tucunduva-Neto, Mauro Saieg, Andre-Luiz Costa, Cláudio de Freitas

**Affiliations:** 1 School of Dentistry, USP (University of São Paulo), São Paulo, SP, Brazil; 2 Tucunduva Clinic, Jundiaí, São Paulo, SP, Brazil; 3 Department of Pathology, School of Medical Sciences, Santa Casa de São Paulo, São Paulo, SP, Brazil; 4 School of Dentistry, UNICID (University of São Paulo City), São Paulo, SP, Brazil

## Abstract

**Background:**

To analyze the face vascularization pattern using B-mode and Doppler ultrasonography, and also propose an arterial vessel mapping.

**Material and Methods:**

The investigation was performed on 20 ultrasonography exams of facial vessels through linear and endocavitary transducers. We analyzed and determined the average values for diameters, peak systolic velocity and resistive index of the following arteries: external carotid, lingual, deep lingual, sublingual, facial, submental, inferior labial, superior labial, angular, maxillary inferior alveolar, mental, buccal, greater palatine, infraorbital, superficial temporal, transverse facial and frontal.

**Results:**

Data was obtained allowing the analysis of the tissue hemodynamics. We were able to map the vascularization of the face and it was possible to access three arteries of small diameter (0,60mm angular artery; 0,55mm greater palatine artery; 0,45mm infraorbital artery).

**Conclusions:**

The results presented in this article are valid tool supporting the non-invasive mapping of facial vascularization.

**Key words:**Anatomy, vascularization, ultrasonography, doppler.

## Introduction

The current diversity of surgical interventions involving the face makes it essential to carry a diagnostic study of the face arterial mapping. Deviation of normal vascular parameters may guide surgical planning and even interfere with patient´s post-operation recovery (1).

The image acquisition methods for investigate vascularization may be expensive and jeopardize the patient’s condition. The ultrasonography is safe and suitable for simple assessment of blood vessels, and also enables a real-time interpretation (2).

In Doppler ultrasonography is possible to observe the anatomic structure by B-mode, arterial and venous phase in the same exam, as well to make consideration on hemodynamic by spatial availability of the vessel (qualitative analysis) and by graphs of Doppler flow velocity waves (1).

Ultrasonography has been used in dentistry mainly in periapical injuries to make differential diagnosis between a granuloma and a cystic lesion (2) or to intraosseous lesions (3,4). Others studies refer to the parotid gland performing Doppler ultrasonography (5,6).

Some anatomical studies of facial vascularization have been published so far (7-15). However, hemodynamic analysis of facial vessels has not been well described yet. In the current study, with the use of ultrasound guided images, we analyzed eighteen different arteries of the face according to their diameter, peak systolic velocity (PSV) and resistive index (RI), in order to determine the average values of these parameters in standard conditions.

## Material and Methods

This study was approved by the Ethics Committee of the University of São Paulo and all participants have signed the informed consent. A single research sonographer, with 21 years of experience, has obtained all the measurements. A total of 20 healthy nonsmokers patients comprised the sample (9 men; 11 women), aged between 20 to 57 years (mean age of 38.5 years), with body mass index from 17.3 to 33.6 (mean of 25.45). All patients had both sides of arteries (right and left of the most bold one spot). We analyzed facial vessels (right and left sides) using B-mode and Doppler, containing lingual arteries and its branches, sublingual artery, deep lingual artery, facial artery and its branches, submental artery, inferior labial artery, superior labial artery, angular artery, maxillary artery and its branches, inferior alveolar artery and its mental branch, buccal artery, greater palatine artery and infra orbital artery; superficial temporal artery and its branches, transverse facial artery and frontal artery.

- Ultrasound-localized technique

The transducer was placed in the longitudinal position; the location of the transducer to assess every artery was the following.

External carotid artery was examined in front of the anterior border of the sternocleidomastoid muscle and below and behind the intermediate tendon of the digastric muscle.

Lingual artery was observed at its origin, above the upper thyroid artery, above the greater horn of the hyoid bone.

deep lingual artery was studied in the middle of dorsum of tongue.

Sublingual artery was found on the mylohyoid muscle in the pre-molars region.

Facial artery was analyzed at the intersection of border of the mandible with the anterior border of the masseter muscle.

Sub mental artery was observed to the lower border of the mandible, below the mylohyoid muscle.

Inferior labial artery was found in its origin, near the emergence of vessel facial artery, before the orbicularis muscle of the lips.

Superior labial artery was also observed in its origin, in the facial artery.

Angular artery was studied in the middle portion of the frontal process of the maxilla, lateral to the nasal pyriform aperture.

Maxillary artery was found behind the ramus of mandible and was measured in the bifurcation of the external carotid artery in its terminal branches.

Inferior alveolar artery was studied just above the lingula of the mandible on the medial surface of the ramus of mandible, laterally to the pterygopalatine rugae.

Mentual artery was seen in the emergence of the mental foramen, between the lower premolars, in the vestibular fornix.

Buccal artery was observed near the region of retromolar area, marginally above the oblique line.

Greater palatine artery was localized in the angle formed between the palatal bone and the alveolar ridge next to last molar upper dental arch.

Infra orbital artery was studied below the infra orbital margin.

Superficial temporal artery was measured behind the condyle, above the zygomatic arch.

Transverse facial artery was seen below and parallel to the zygomatic arch, close to its origin.

Frontal artery was studied in parallel with an oblique line that followed the external orbital process the external auditory pore.

**Figure 1:**
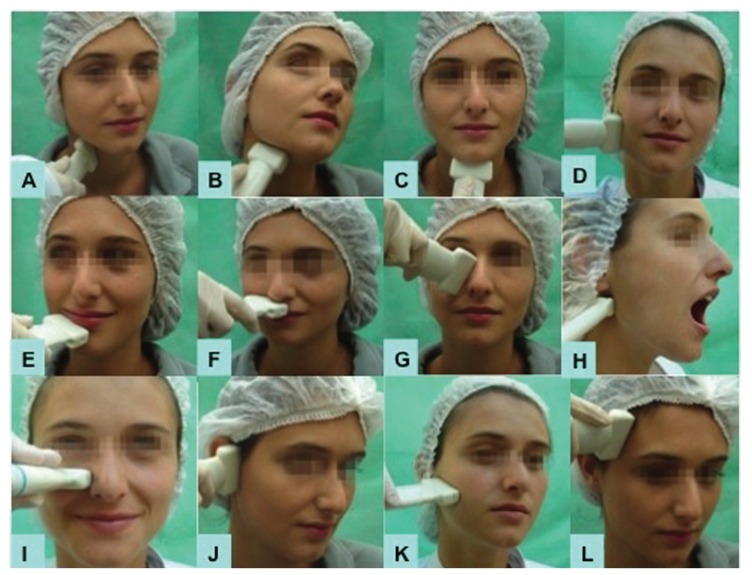
Extraoral technique used for the assessment of: A-external carotid a., B- lingual a., C- submental a., D- facial a., E- inferior labial a., F- superior labial a., G- angular a., H- maxillary a., I- infraorbital a., J- temporal superficial a., K- transverse facial a., L-frontal a.

**Figure 2:**
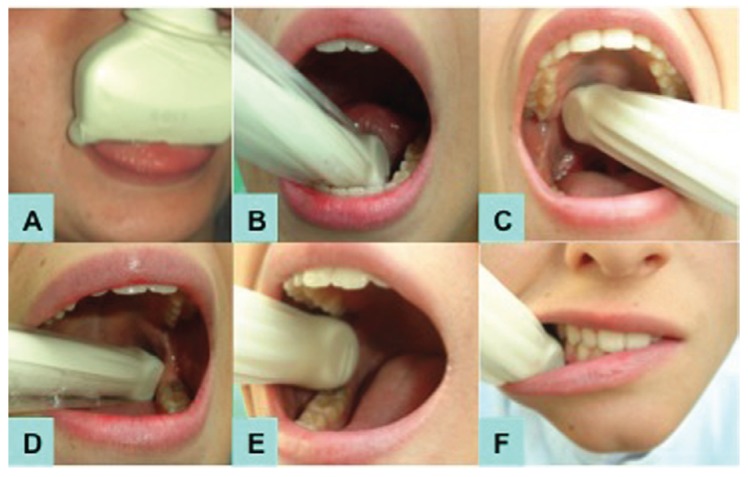
Intraoral technique used for the assessment of: A- deep lingual a., B- sublingual a., C- greater palatine a., D- inferior alveolar a., E- bucal a., F- mental a.

Figures 1,2 shows positioning of the transducer to explore each artery.

The images were obtained using a portable Terason t3000 (Teratech Corporation, Burlington, MA, USA), a 5-12MHz linear transducer model 12L5 and 4-8MHz endocavitary transducer model 8EC4. We used in extraoral vessels a thin layer of gel. An intraoral gel was applied between the transducer and the latex protection, and between the protector and the mucosa was only saliva. First the extraoral vessels were studied using the linear transducer, and last the deep lingual artery was assessed with the same transducer.

Right vessels and then left vessels were observed from lower to upper direction. After that, we studied the maxillary artery (extraoral access) and the intraoral vessels using the endocavitary transducer. The transducer´s long axis was placed parallel to large vessels. Doppler analysis was generated using the standard angle from 0 to 60º adjusted to the blood flow. Doppler sample volume was 1.5 to 3.00. The pulse repetition frequency and the wall filter were regulated to avoid artifacts. Figures 1,2 demonstrate the assessment of arteries Doppler sonography.

We used a color Doppler to large vessels and amplitude Doppler to smaller vessels without considering the insonation angle in this case. Data regarding arteries diameter and graphs of flow velocity waves, PSV and RI, was collected.

## Results

We obtained a total of 40 exams (all patients had both sides of arteries examined). The mean values and ranges obtained are shown in  Table 1.

**Table 1:**
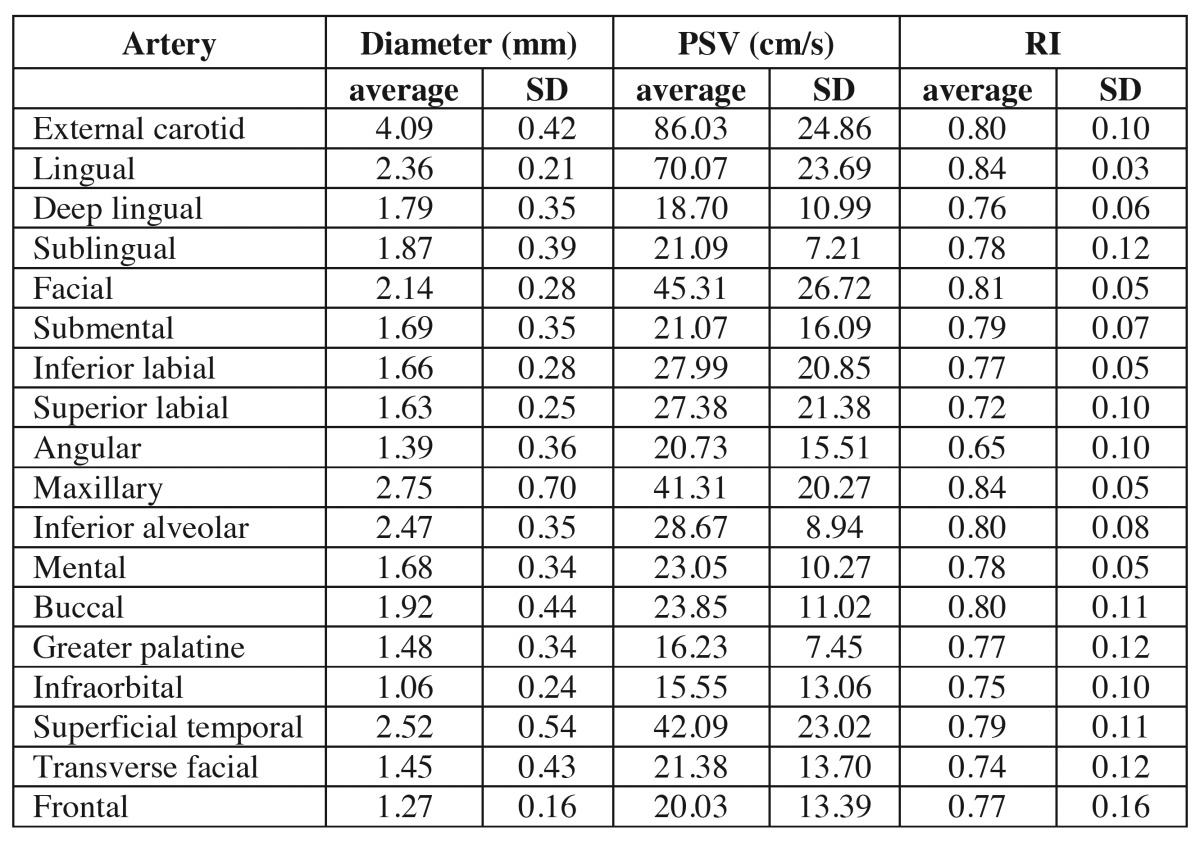
Arteries, diameters, peak systolic velocity (PSV) and resistive index (RI).

The external carotid artery showed an average diameter of 4,09mm (deviation of 0.42), PSV of 86 (SD 24.86) and RI 0.80 (SD 0.10), the highest values.

The infra orbital artery showed an average diameter of 1.06mm (SD 0.24), PSV of 15.55 (SD 13.06) and IR 0.75 (SD 0.10), the lowest values.

We found that the greater palatine artery had lower detection and was observed 18 times in the right side and 17 times in the left side. All arteries were able to provide diameter parameters, PSV and RI, using a popular endocavitary transducer.

**Figure 3:**
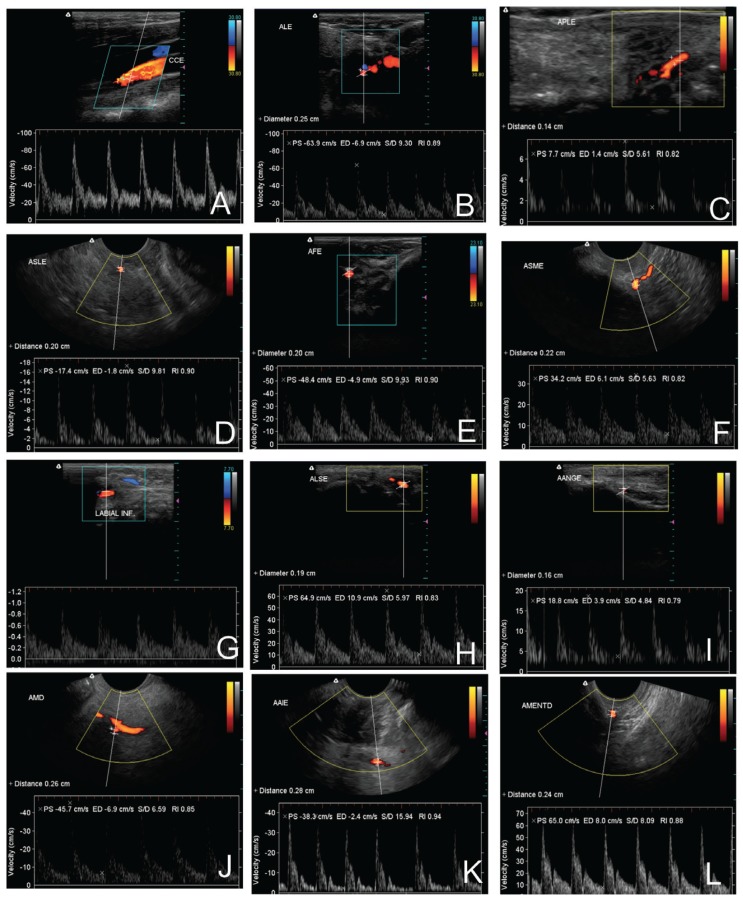
Ultrasonographies of the extraoral arteries: A-external carotid a., B- lingual a., C- submental a., D- facial a., E- inferior labial a., F- superior labial a., G- angular a., H- maxillary a., I- infraorbital a., J- temporal superficial a., K- transverse facial a., L-frontal a.

**Figure 4:**
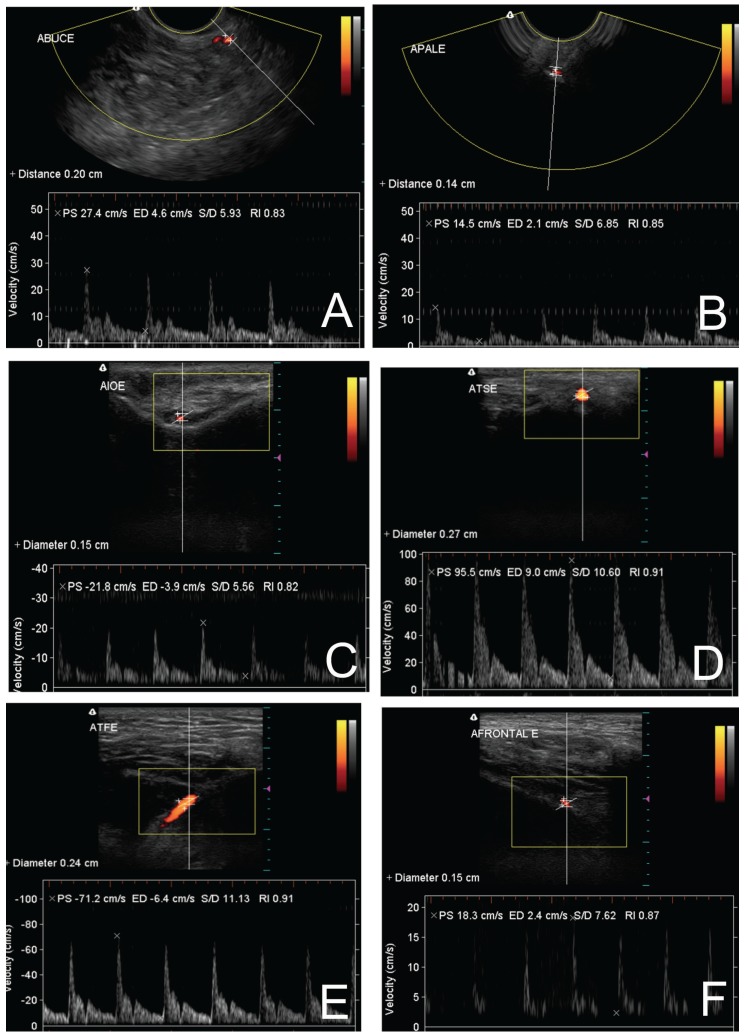
Ultrasonographies of the intraoral arteries: A- deep lingual a., B- sublingual a., C- greater palatine a., D- inferior alveolar a., E- bucal a., F- mental a.

Figures 3,4 shows ultrasonographies of the extraoral and intraoral arteries, respectively.

## Discussion

The blood supply of a region is critical to the health of tissues (6). Therefore, it is essential to know the characteristics of the vascularity of a particular area, either before a surgical procedure, or in the follow-up after a postoperative intervention, or any other situation that would lead to a reduction of irrigation (7). To our knowledge, there is no published study evaluating a complete vascularization of the face.

Doppler sonography is the method that enables the analysis of total blood flow characteristics in the target organ, including both arterial and venous flow (13,16). In this study, we demonstrated characteristics of a map of facial areas using Doppler sonography.

The vascular supply to the face is anastomosis network (15). The sublingual, submental, inferior and mental labial arteries join with the anteroinferior portion of the face; also the superior, infraorbital and transverse facial and bucal arteries join with the transition of third medium to lower part of the face (12).

In palate region the greater palatine meets the sphenopalatine arteries. In the medium plane the facial branches may suffer anastomosis with contralateral branches (15). The angular and frontal arteries are combined with branches from internal carotid artery, which promote the vascularization of the upper portion of the orbit its content and the forehead. Such vessels have reduced diameter, and information about them in literature are rare.

The study of facial vessels arises in regular and intern carotid arteries (15). The exams recommended to study these vessels compose the conventional angiography, computed tomography, magnetic resonance imaging within time-of-flight technique or phase-contrast (1,17).

Risks of angiography are highlighted many times in the literature (1,14,17) including the possibility of injuries to the vessel and its consequences. Other exceptions are presented in the literature on adverse effects of the use of contrast. Only two exams currently available do not used contrast, the magnetic resonance imaging through time-of-flight technique and the Doppler ultrasonography. Bialek *et al*. (3) emphasized that differently of other methods for imaging of vessels, the ultrasonography enables the analysis of soft parts and bone surfaces using the same transducer simultaneously (B-mode and Doppler).

When vessels’ size is considered the degree of observation of medium and small vessel is discussed. Nagase *et al*. (12) analyzed on Doppler ultrasonography the facial artery in two regions close to mandibular basis in front of the masseter muscle and close to the labial commissure. In the region next to mandibular basis they obtained mean diameter of 2.7mm. Another study observed the same artery and found diameter of 2.6mm. In our analysis, the facial artery had a mean diameter of 2.14mm.

Some authors (8,9) observed several arteries (inferior alveolar artery, mental artery, sublingual artery, inferior labial artery, submental artery, greater palatine artery and sphenopalatine artery) using an 8MHz pencil probe specially design for this aim.

Vazquez *et al*. (18) followed the trajectory of the inferior labial artery in patients with caliber-persistence of this artery. Although values were not reported, the authors stated that graphs of flow velocity waves did not show differences of regular pattern, despite these arteries remained constant in diameter. Jacobovicz *et al*. (10) reinforced the use of RI because this is an index that estimates peripheral resistance of blood flow, enabling a hemodynamic analysis, highlighting the fact that PSV at medium vessels may be high in the presence of stenosis.

We verified the difficult to obtain a PSV with less difference between maximum and minimal values, particularly because sometimes the ideal angulation to analyze the vessels is not obtained for the rigid bone surface, where these vessels are placed (bucal artery, greater palatine artery, angular artery and infra orbital artery), and could in some cases be closed to angle of 90º, which would avoid the screening of the vessel. It is important to say that we used color Doppler to observe large vessels and amplitude Doppler for small vessels, which is more sensible. The use of both techniques did not cause changes to studied vessels in diameter values, RI or PSV.

Eiseman *et al*. (8) reported an inverted flow of lingual artery through sublingual branch, facial artery by its segmental branches and lower lip and mylohyoid artery. Despite in this study, the reversed flow was not observed in any artery, the detection of the described vessels and other vessels important to anastomosis, either anteroposterior or contra lateral direction, reinforces the concept of vascular mapping. It also enables to evaluate possibilities of blood supply in the case of surgical occlusion or vascular disease like atheromatosis. Such situations would cause, according to the same authors, a deficiency of blood supply and as a consequence the atrophy of structures that irrigate arteries territory. In our study, the inverted flow was not recorded in vessels assessed by color Doppler. The amplitude Doppler does not detect the blood flow direction.

Notwithstanding the diameter of some arteries is described in the literature, most of values measured (19,20) were obtained from arteries of cadavers, which was prior formaldehyde-fixed and then injected with latex. These procedures may cause dehydration of the vessel and morphologic changes due to latex injection. In ultrasonography the diameter of blood flow depicted through color Doppler mapping was measured corresponding to the inner vessel.

PVS was one of parameters that showed the greatest variation, especially in smaller diameter vessels. This is probably due to VPS is a parameter given in the Doppler scan, and this in turn receives echoes of the blood cells moving inside the vessel. Assuming that the blood elements more centralized in the lumen have a shift faster than those near the wall, which may suffer friction arising from contact with this, and thus to reduction in speed.

After state this exception we can confirm that these findings are in agreement with studies in the literature. *Edizer et al*. (19) found inferior labial artery of 1.2mm, and Pinar *et al*. (20) pointed in their study an inferior labial artery of 1.0mm. In this study, this artery measured 1.66mm. Jiang *et al*. (9) using computed tomography reported a facial artery of 2.83mm at the same site we had evaluated in our study. Pinar *et al*. (20) indicated a superior labial artery of 1.6mm and, in our study, we found 1.63mm around the facial artery. The values were closed and differences should be due to methods used and specific area analyzed.

Because in this analysis patients were healthy and did not have any surgical procedure, in some of them a particular vessel could not be seen in all exams. Therefore, we could not found anatomic variants in sublingual artery, superior labial artery, angular artery, superficial temporal artery, transverse facial artery and superior labial artery like those variants described by others authors (16,19,20).

In addition, related to other vessels that we could not detect in 100% of cases as bucal artery, greater palatine artery and infraorbital artery, no data in literature was found. So, a further study with more focus on trajectory of particular vessel is therefore suggested. In light of the results shown in this research, our work provides a detailed description of facial vascularization, diameter parameters, PSV and RI that enabled hemodynamic analysis of vessels. This knowledge is helpful not only for dentists, but also for plastic surgeons, and other physicians for preoperative planning and intraoperative management.
